# Evaluation of Potential Protective Factors Against Metabolic Syndrome in Bottlenose Dolphins: Feeding and Activity Patterns of Dolphins in Sarasota Bay, Florida

**DOI:** 10.3389/fendo.2013.00139

**Published:** 2013-10-10

**Authors:** Randall S. Wells, Katherine A. McHugh, David C. Douglas, Steve Shippee, Elizabeth Berens McCabe, Nélio B. Barros, Goldie T. Phillips

**Affiliations:** ^1^Sarasota Dolphins Research Program, Chicago Zoological Society, c/o Mote Marine Laboratory, Sarasota, FL, USA; ^2^U.S. Geological Survey Alaska Science Center, Juneau, AK, USA; ^3^Physiological Ecology and Bioenergetics Laboratory, Department of Biology, University of Central Florida, Orlando, FL, USA; ^4^Duke University Marine Laboratory, Beaufort, NC, USA

**Keywords:** metabolic disease, diet, age, bottlenose dolphin, activity cycles, foraging, telemetry

## Abstract

Free-ranging bottlenose dolphins (*Tursiops truncatus*) living in Sarasota Bay, Florida appear to have a lower risk of developing insulin resistance and metabolic syndrome compared to a group of dolphins managed under human care. Similar to humans, differences in diet and activity cycles between these groups may explain why Sarasota dolphins have lower insulin, glucose, and lipids. To identify potential protective factors against metabolic syndrome, existing and new data were incorporated to describe feeding and activity patterns of the Sarasota Bay wild dolphin community. Sarasota dolphins eat a wide variety of live fish and spend 10–20% of daylight hours foraging and feeding. Feeding occurs throughout the day, with the dolphins eating small proportions of their total daily intake in brief bouts. The natural pattern of wild dolphins is to feed as necessary and possible at any time of the day or night. Wild dolphins rarely eat dead fish or consume large amounts of prey in concentrated time periods. Wild dolphins are active throughout the day and night; they may engage in bouts of each key activity category at any time during daytime. Dive patterns of radio-tagged dolphins varied only slightly with time of day. Travel rates may be slightly lower at night, suggesting a diurnal rhythm, albeit not one involving complete, extended rest. In comparison, the managed dolphins are older; often fed a smaller variety of frozen-thawed fish types; fed fish species not in their natural diet; feedings and engaged activities are often during the day; and they are fed larger but fewer meals. In summary, potential protective factors against metabolic syndrome in dolphins may include young age, activity, and small meals fed throughout the day and night, and specific fish nutrients. These protective factors against insulin resistance and type 2 diabetes are similar to those reported in humans. Further studies may benefit humans and dolphins.

## Introduction

Insulin resistance and metabolic syndrome have been identified in a group of bottlenose dolphins managed under human care (*Tursiops truncatus*) (1, 2). While dolphins do not appear to progress to type 2 diabetes, they can develop chronic hyperlipidemia, postprandial hyperinsulinemia, and fatty liver disease (2, 3). To identify populations at higher and lower risk of metabolic syndrome, metabolic blood values were compared between this well-studied managed collection and another well-studied group of free-ranging dolphins living in Sarasota Bay, Florida (4). Sarasota Bay dolphins had lower postprandial insulin, glucose, triglycerides, cholesterol, and liver enzymes, supporting that Sarasota Bay dolphins have a lower risk of insulin resistance and metabolic syndrome.

In humans, advanced age, large meals, lack of key fish-based nutrients (e.g., n-3 fatty acids), and abnormal work schedules or activity levels have been reported as known or potential risk factors for insulin resistance and metabolic syndrome (5–9). Similarly, it has been hypothesized that reasons for insulin resistance in the managed dolphin population might be related to the facts that: (1) managed populations are, on average, older than free-ranging populations, (2) they may have larger but fewer meals than their free-ranging counterparts, (3) there may be nutritional differences between natural prey and commercially available food provided to the managed population, and (4) activities and work schedules of the managed dolphins may vary from their natural circadian rhythm (3). Thus, understanding diets and feeding behaviors, activity levels, and day/night activity of Sarasota Bay dolphins could provide insights into why Sarasota Bay dolphins appear to have a low risk of developing insulin resistance and metabolic syndrome.

Very few situations exist where wild bottlenose dolphin activity levels and day/night patterns can be studied within a known context of diet and feeding behaviors. The long-term “Natural Laboratory” situation in Sarasota Bay, Florida, provides unique opportunities to study the ecology and behavior of a well-known resident community of bottlenose dolphins (10–12). Research conducted in and around Sarasota Bay since 1970 has demonstrated the existence of multi-decadal, multi-generational, year-round residency by a community of recognizable individual dolphins living within a well-defined home range, set within a mosaic of adjacent dolphin communities along the Florida coast. The Sarasota community’s range is largely composed of sheltered, shallow waters that facilitate a variety of kinds of field research. Taken together, the accessible study area and predictable presence of a well-known cast of characters for which detailed long-term background is available creates a unique situation for dolphin behavioral and ecological research under natural circumstances.

A number of approaches and tools have been developed or adapted over the years to enhance our understanding of the lives of the resident dolphins of Sarasota Bay (12). It has been possible to learn about the diets of local dolphins, the availability of their prey, and behaviors used by the dolphins to capture prey. Direct behavioral observations of well-known residents have provided quantitative data on activity patterns. Electronic tags have enabled researchers to follow individuals around the clock, increasing opportunities for direct observations and providing indirect measures of movements and activities. Telemetry has made it possible to identify the occurrence of dolphin feeding events even when the animals are not visible to the researcher. Acoustic recordings have provided information on activity levels and the occurrence of specific activities such as feeding via its connection to echolocation, filling knowledge gaps about what the dolphins do during the night and when they are out of sight. Taken together, the findings from these independent lines of research involving a single community of wild dolphins can begin to address questions related to understanding the occurrence of the dolphin’s natural diabetes-like states and development of insulin resistance and metabolic syndrome. Identifying risk and protective factors may help prevent and treat insulin resistance and metabolic syndrome in both dolphins and humans.

## Methods and Materials

### Study area and dolphin population

**Figure 1:**
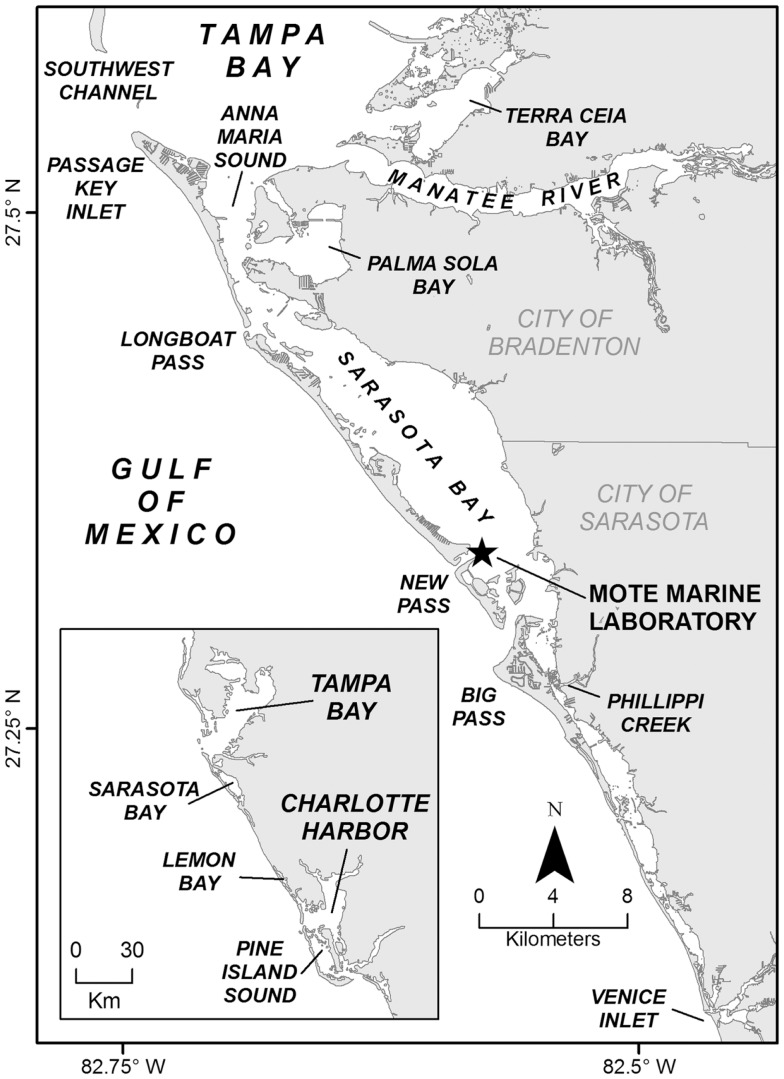
**Long-term study area of the Sarasota Dolphin Research Program along the central west coast of Florida**. Field base at Mote Marine Laboratory indicated.

As of 2013, about 160 dolphins, spanning at least four concurrent generations, made up the long-term resident Sarasota Bay dolphin community. These dolphins reside in the inshore waters from southern Tampa Bay, including Passage Key Inlet, Terra Ceia Bay, and the Manatee River, southward to about Venice Inlet (Figure 1). This estuarine region includes sheltered, shallow bays (up to 4 m deep) connected by channels (up to 3 m deep), and connecting with the Gulf of Mexico through narrow passes (up to 10 m deep) between a string of barrier islands. Inshore of the barrier islands, habitats include seagrass meadows, unvegetated bay bottom, rivers and creeks, and sand flats. Shorelines are mostly developed and unvegetated, but some mangrove fringing forests remain, typically bordering seagrass meadows. Nearshore Gulf waters provide sand bottom habitat, ranging up to about 6 m deep within the typical range of the Sarasota Bay dolphins, up to several kilometer offshore. In the Gulf, Sarasota dolphins typically concentrated around the passes (13, 14).

Approximately 96% of the animals using Sarasota Bay and associated waters on a regular basis are individually identifiable from natural markings and/or from markings applied during brief capture-release events for health assessment, life history, tagging, ecological, and behavioral research (11, 12, 15). Two of the 12 dolphins first tagged in 1970–1971 were still observed in the area in 2013. Systematic photographic identification (photo-ID) surveys are conducted monthly around Sarasota Bay to document dolphin abundance and distribution in the region. The current photo-ID catalog for the central west coast of Florida includes more than 4,800 individuals. These identifiable individuals have been re-identified more than 113,000 times in the more than 41,000 dolphin groups recorded since 1970. Some individuals have been observed more than 1,400 times over more than four decades.

### Age

Ages of free-ranging dolphins were determined in one of two ways. In most cases, dolphins were of known age because they were first observed as young calves with well-known resident mothers (12). For the remainder, age was estimated from examination of growth layer groups in a tooth extracted under local anesthesia during capture-release operations, or from carcasses upon necropsy (16).

### Dolphin feeding and activity patterns

Dolphin activity patterns in Sarasota Bay have been quantified through focal animal behavioral observations, as pioneered by Altmann (17). More than 2,300 focal animal behavioral follows have been performed with Sarasota Bay dolphins since 1989. Observations of day-time feeding were obtained both from small boats and through use of overhead video (18–20). Continuous behavioral observations when dolphins were above and below the surface were possible in shallow water via a video camera suspended below a 10-m-long helium filled aerostat, operating 50 m above the water. The aerostat was tethered to a 6-m outboard powered vessel, and hydrophones were towed from the vessel for concurrent acoustic recordings.

**Figure 2:**
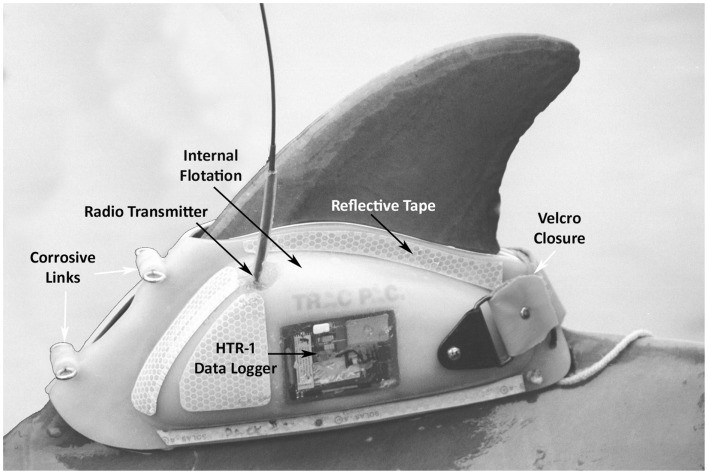
**Trac Pac tag attached to dolphin dorsal fin**. HTR-1 Data Logger (Wildlife Computers) recorded stomach temperatures from the telemeter pill; Radio Transmitter (Advanced Telemetry Systems) emitted 150 MHz pings, receivable from 1 to 3 km distance for up to 90 days; Internal Flotation embedded within plastic kept the pack afloat at surface after jettisoning with the antenna oriented above water; Corrosive Links composed of zinc barrel and steel eyebolts were size-selected to dissolve between 8 and 24 h; Reflective Tape allowed observers to detect the animal at night using a spot light; Velcro Closure used to compress trailing edge of pack on the fin keeping suction cup liner firmly in place until pack separated at front.

**Figure 3:**
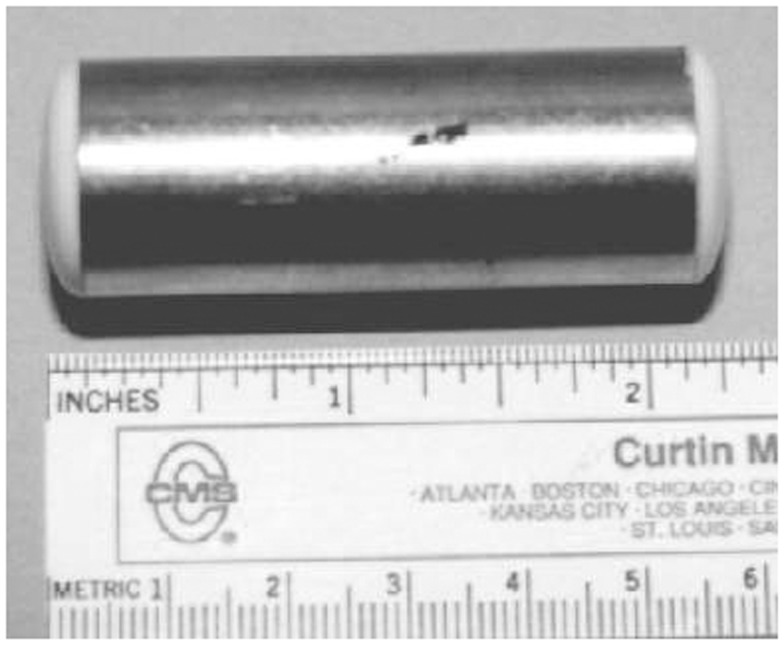
**Forestomach temperature measuring telemetry pill**.

Shippee et al. (21) observed day and night feeding behavior while tracking Sarasota Bay dolphins tagged with a molded thermoplastic saddle (Trac Pac, Inc., Fort Walton Beach, FL, USA) held in place on the dorsal fin by bath-mat style suction cups, a Velcro fastener, and a timed corrosible link. The Trac Pac was attached to the dolphins during capture-release for health assessment, just prior to release. The device housed several archival data recorders, including a swim speed datalogger, manufactured by Wildlife Computers (Redmond, WA, USA) (Figure 2). An inert metal, 5.5 cm × 2 cm, 50 g temperature telemeter pill (Wildlife Computers STP) was inserted via esophageal tube into the dolphin’s forestomach (Figure 3). The STP transmitted 5 kHz radio pulses at a rate that varied predictably with changes in the temperature of the pill casing. The telemetered signal was received by an HTR-1 data logger on the Trac Pac that was programed to record stomach temperature readings every 15 s. Rapid declines of 0.5°C > 3.0°C in forestomach temperature (FST) were assumed to indicate prey ingestion. It was expected that the stomach temperature pill would be vomited by the dolphin within 2 days (average retention time was 30 h based on previous work (21). Following release, the tagged dolphin was tracked via VHF tag by observers on a 5 m outboard boat for the duration of the tag attachment lasting from 2 to over 24 h. The tag jettisoned from the dolphin after corrosion of two metal links, and was then recovered for data download.

Foraging was defined by two methods: (1) when an animal was observed to be actively engaged in feeding (splashing, fast swimming, fish jumping, etc.), and (2) from changes in FST as recorded by the data logger on the Trac Pac. When a tagged animal was out of sight (e.g., at night) undifferentiated observations of foraging/socializing were noted based on location and whether the animal was alone or in a group. FSTs and associated movements and behavior were recorded for seven of the eight Sarasota Bay dolphins tagged with Trac Pacs during 2000–2006, with attachment durations of up to 41 h (21).

In addition to collecting foraging observations, Shippee et al. (21) tracked and observed the general activities of two Sarasota Bay dolphins tagged with Trac Pacs through both day and night. An automatic direction finding radio-tracking system facilitated keeping the observation vessel within 100 m of the tagged dolphin even at night. In addition to the movement data derived from radio-tracking, general observations of activities at night were often possible with available light. On occasion, a spotlight was used to confirm dolphin identification from shine off reflective tape on the tag, but the spotlight was used sparingly and no obvious effects were noted. Transit swimming was defined as steady directional swimming over a protracted distance (usually >1 km). Resting, or sleep-like activity, was identified as slow-speed, constant-direction swimming with rhythmic respiration rates and relaxed exhalations (22).

An observational study by Waples (23) during 1992–1994 was focused specifically on quantifying activity patterns relative to energetics. Dolphins were located by radio-tracking of VHF tags and by surveying from a 5 to 6-m outboard research vessel throughout the study area while continually scanning for dolphins. Individual dolphins were identified by dorsal fin markings, and prior to initiating focal animal behavioral observations, data were collected on time and location, group size, environmental and sightability conditions, and general dolphin activity. Instantaneous sampling methods were used to collect data on focal activity at 3-min sample intervals (17). At each sample point, the activity of the focal animal was recorded. The key activities recorded by Waples and most other researchers working with Sarasota Bay dolphins [e.g., (24)] included:

Travel – directed movement either in a straight line or zig zag.
Mill – non-directed movement.
Probable feed – strong indication of feeding without positive confirmation of the presence of a captured prey item; includes sub-surface swirling, fish leaping, and dolphins chasing fish.
Feed – direct evidence of feeding, i.e., a fish in the mouth of the focal animal.
Socialize – tactile or active interaction with at least one other dolphin.
Rest – slow quiescent activity or remaining motionless at the water surface.
Play – interaction with an object, i.e., algae, stingrays, or boats.

Acoustic activity was monitored in two ways, via deployments of digital acoustic recording tags (DTAGs), and through deployment of a fixed hydrophone array in one of the bays used heavily by Sarasota dolphins. Short-term tag attachments have provided a variety of data on Sarasota Bay dolphins, through attachment of onboard computers via suction cups, and tracking via VHF tags of the animals during tag deployment and recovery of the tags. DTAGs have been developed and tested with Sarasota Bay dolphins in dozens of deployments since 1990 (25–27). Early versions, attached to the dorsal fin, remained attached for a few hours; recent versions attached to dolphins’ backs, have remained attached for 24 h, providing around-the-clock data on acoustics and behavioral parameters (P. L. Tyack, personal communication).

The occurrence of dolphin sounds during the day and night was also documented by deploying an autonomous acoustic recorder (DSG-Ocean, Loggerhead Instruments; system sensitivity, −160.5 dB re 1 V/μPa) in an area of high dolphin density. The recorder was placed on the sea floor at a depth of 1.3 m depth. Recordings were collected during 4–11 September 2012 (Phillips, unpublished). The 50 kHz sampling rate of the recorder was insufficient to record all the frequencies contained within echolocation clicks. However, recordings were sufficient for the examination of click occurrence. The template detector in the sound analysis software Extensible Bioacoustic Tool was used for click detection. A correlation threshold value of 0.4 was used, as this value minimized false detections while detecting at least some of the actual clicks, when tested with a sample of this dataset. Detector performance was assessed via the manual examination of 30 10 min recording samples randomly selected from the entire dataset, and these results were used to produce final click abundance estimates reflecting false positive and false negative rates.

### Dolphin diet

Dolphin prey items were identified and quantified from the stomach contents of 33 dolphins with documented sighting histories from the Sarasota area, recovered by Mote Marine Laboratory’s Stranding Investigations Program as carcasses along the central west coast of Florida during 1984–2006. Stomachs of stranded dolphins were removed during necropsy and frozen for use in stomach content analysis. Contents from all stomach chambers were removed, sorted, and prey items were identified to the lowest possible taxon, usually to species but in some cases it was only possible to identify prey to the family level. All available stomach content data for Sarasota Bay dolphins are summarized here, including data published previously by Barros and Wells (28) and Berens McCabe et al. (29). The applicability of stomach content data for describing the diet of live, free-ranging Sarasota Bay dolphins was validated through DNA analyses of gastric samples and feces by Dunshea et al. (30).

Prey availability has been assessed since 2004 from seasonal multispecies fisheries surveys, using a 183-m × 6.7-m purse seine with 2.5-cm mesh (29, 31). The net is deployed from a 9-m flat-bottomed skiff in waters between 0.4 and 4 m deep. The net samples the entire water column, from surface to bottom. The fish sampling study area includes most of the range of the resident dolphins, encompassing the estuarine waters from Passage Key Inlet at the southwestern edge of Tampa Bay (27.55°N/82.74°W) southward to Phillippi Creek, south of Sarasota Bay (27.27°N/82.53°W) (Figure 1). All fishes, cephalopods, and penaeid shrimps caught in each purse seine set are identified and counted. Catch per unit effort, or the number of organisms caught per standardized purse seine set, is used as an index of prey availability (29). Prey selectivity is determined by comparing the proportion of numerical prey abundance of fish species in stomach contents relative to the availability of that species in the Sarasota Bay study area using standardized forage ratios (29).

### Dolphin movements and dive patterns

Movement patterns of Sarasota Bay dolphins across day and night hours have been determined from radio-tracking since 1975. During 1975–1976, UHF tags were attached to the dorsal fins of 10 dolphins to try to define ranging patterns for dolphins in the Sarasota Bay area (13). These were tracked from 8 m long power and sailboats via automatic direction finder for up to 22 days. Small VHF tags were attached to cattle ear tags (roto tags) and deployed on dolphins during 1992–1994 for a study of dolphin energetics (23). These tags were removed after 3–4 days. During 2000–2006, eight dolphins were tracked by VHF transmitters associated with short-term Trac Pacs (21). Tracking of VHF tags requires line-of-sight contact, and direct radio-tracking was accomplished from various 6 to 8 m long vessels.

**Figure 4:**
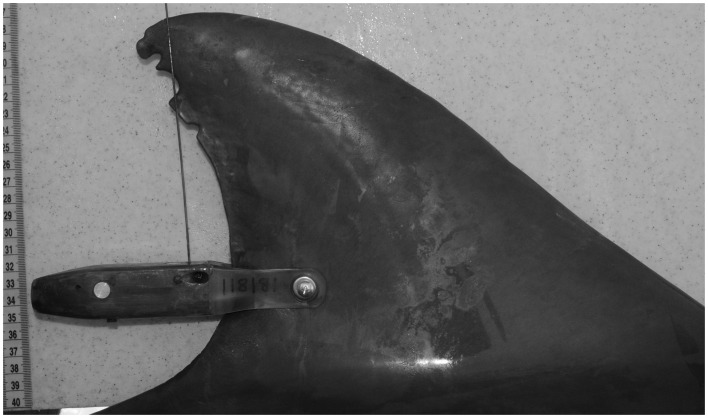
**Satellite-linked time-depth-recording transmitter (Wildlife Computers SPLASH tag) attached to adult female bottlenose dolphin F113 in May 2012**.

Remote tracking was accomplished by means of satellite-linked tags. An early version of a satellite-linked time-depth-recording tag (TDR) was deployed on an adult female in 1990 along the northern edge of the Sarasota dolphin home range, and the individual was tracked through Tampa Bay for 25 days (32). Wells et al. (unpublished) conducted tests in 2012 of a much smaller satellite-linked TDR tag (SPLASH, Wildlife Computers, Redmond, WA, USA), attached by means of a single pin to the trailing edge of the dorsal fin (Figure 4). Three SPLASH tags were deployed during 2012 on adult dolphins (Table 1) and were tracked for 73–100 days. The duty cycles were set to optimize access to satellites as well as to provide locations at various (local) times during the day: 03:00–04:59, 09:00–11:59, and 20:00–22:59. Dive duration data were recorded throughout the day, and provided as numbers of dives occurring within specific time periods (bins) during four 6-h time-of-day categories: Dawn (04:00–09:59), Day (10:00–15:59), Dusk (16:00–21:59), and Night (22:00–03:59). Location data were filtered on the basis of Argos location class errors, with only the highest quality locations being used for plotting and analyses (LC 3, 2, 1).

**Table 1:** **Sarasota Bay dolphins tagged with satellite-linked transmitters in May 2012**.

Dolphin	Deploy date	Sex	Age (years)	Length (cm)	Weight (kg)	No. tracking days	No. follows	Total Observation time (min)
F113	May 07	F	16	240	157	75	7	222
FB20	May 07	M	23	273	300	73	6	360
F242	May 08	M	22	281	257	100	6	327

Travel rates were determined through direct tracking, for example with the Trac Pacs containing swim speed data loggers, by measuring distance between known locations during boat based focal follows, and through calculations from remote tracking data. The CRAWL method [continuous-time correlated random walk (33)] was applied to location data from the three dolphins affixed with satellite-linked TDR tags in 2012. This method estimated the location of a dolphin every 60 min, based on the Argos locations and their LC, allowing distances and movement rates to be estimated over uniform time intervals.

## Results

### Age

In 2012, the most recent full year of data collection in Sarasota Bay, 95% of the Sarasota Bay resident dolphins were of known age, and the others could be assigned to age classes relative to maturity. As of 2013, the oldest female was 63 years old, and the oldest male was 50 years old. All age classes were represented in the Sarasota Bay population, including 11 calves born in 2012.

### Feeding and activity patterns

Direct observations of bottlenose dolphins in Sarasota Bay have shown that they are active throughout the day, interspersing bouts of different kinds of activity. Overall, Waples (23) found that Sarasota Bay dolphins spent 67% of daylight hours traveling, 14% milling, 13% feeding, 4% socializing, and 2% resting. There were no seasonal differences among the combined data, but significant seasonal differences emerged when the data were examined by sex. Females spent more time feeding and socializing in summer than in winter and spent more time traveling in winter. Males socialized and rested more in winter than in summer and traveled less in winter. Comparisons across the sexes by season found a significant difference only in summer, when males traveled more than females, while females fed, socialized, and rested more than males.

Waples (23) examined activity patterns relative to time of day for Sarasota Bay dolphins during 2-h blocks: morning (09:00–10:59); mid-day (11:00–12:59); early afternoon (13:00–14:59); late afternoon (15:00–16:59), and evening (17:00–18:59). With the exception of rest, all activities occurred across all time periods. Time spent in each activity state ranged from 63 to 76% traveling, 9–18% milling, 9–20% feeding, 2–7% socializing, and 0–3% resting. Across the entire year, peaks in feeding were found in morning and late afternoon, socializing peaked in morning and late afternoon, resting occurred during mid-day through late afternoon, and travel occurred consistently throughout the day. In summer, feeding peaked in morning and late afternoon, but only a single early afternoon peak in socializing was observed; in winter, socializing peaked in morning and early afternoon. In contrast to the overall and summer patterns, winter feeding increased throughout the day, peaking in evening.

Using similar methods to Waples (23), McHugh (24) focused on juvenile Sarasota Bay dolphins and found no significant differences in overall activity budgets by time of day during daylight hours with all activities occurring across all time periods. Activity patterns varied seasonally, however, with less time overall spent foraging in winter and significant diurnal variation in foraging patterns in summer, but not winter. Juvenile foraging in summer occurred primarily in morning and mid-day, declining toward evening, with more traveling later in the day. No other activities varied significantly with time of day.

**Figure 5:**
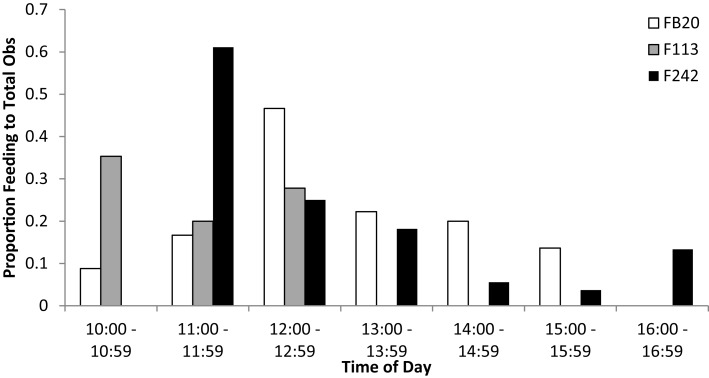
**Occurrence of day-time feeding observations for Sarasota Bay bottlenose dolphins tagged with satellite-linked transmitters in 2012**. Bars indicate the proportion of total focal animal behavioral observations that involved feeding or probable feeding, relative to time of day. Note that no observations of any kind were obtained for dolphin F113 during 1400–1700.

The three Sarasota Bay dolphins tagged with satellite-linked transmitters during spring/summer 2012 were also the subjects of focal animal behavioral observations. Although observations were limited to the period 10:00–17:00, feeding was noted during all available observation periods. Peaks in foraging behavior were observed during morning through mid-day, each dolphin with a different hourly peak from 10:00 to 12:59 (Figure 5), comparable to the timing reported by McHugh (24).

Data from several sources suggest Sarasota Bay dolphins are active at night as well as during the day. In addition to the indirect data on movements and dive patterns from remote radio-tracking described above, direct tracking of dolphins with Trac Pacs provided activity data from around the clock. Three dolphins, females F185 and F189, and male F100, were tracked during day and night. F185 was tracked for 16.1 h over June 3–4, 2004 (9.5 h at night), F189 was tracked for 41.2 h over February 4–6, 2005 (12.3 h at night), and F100 was tracked for 10 h on June 8, 2006 (3.5 h at night). Shippee et al. (21) identified three classes of activity: transit, forage-socializing, and resting, and all of these activities were observed at intervals both during the day and at night for all three dolphins. Overall, the dolphins spent 47–49% of their time in transit (traveling), 36–45% of their time feeding/socializing, and 7–18% of their time engaged in sleep-like activity. While the absolute proportions differ, the relative frequency of occurrence of these activities is comparable to those reported by Waples (23) and McHugh (24). Resting was observed intermittently and only when the animals were transiting from one activity location to another, especially at times when there was reduced boat activity and tranquil water. On at least one occasion, sleep swimming was interrupted when the animals suddenly began to engage in apparent feeding.

**Figure 6:**
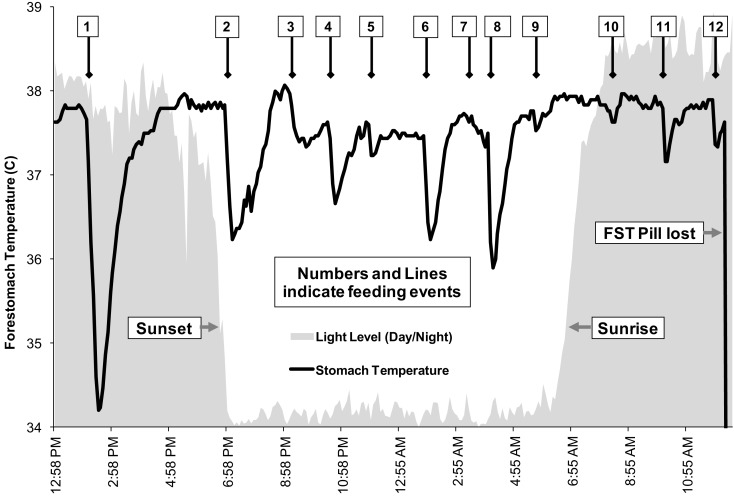
**Foraging record for dolphin F189 as indicated by changes in forestomach temperature**. Twelve apparent feeding events are indicated by arrows, occurring throughout the day and night [from Ref. (21)].

Apparent feeding was identified from FST measures for all three dolphins (21). Over 11 h of telemeter pill retention, one change in stomach temperature indicative of apparent feeding was recorded for F185, a 4-year-old female, and this occurred at night. Direct observations during tracking, along with FST measurements, indicated that F185 engaged in foraging activity during both day and night. Over at least 10 h of telemeter pill retention, 17-year-old male F100 showed two feeding events, one of these at night. During 24 h of telemeter pill retention, 12 apparent feeding events were observed for adult female F189, a 32-year-old female (Figure 6). Her feeding events were relatively evenly distributed throughout the 24-h track, regardless of time of day. During the track of F189, numerous sounds suspected to be from silver perch (*Bairdiella chrysura*) and from Gulf toadfish were recorded at locations where the dolphin was seen feeding in close proximity to the tracking boat (21). The sounds of these fish were more prominent during the evening hours than during daytime, and coincided with increased foraging activity by F189 and her associates. In addition, several stomach temperature changes in the FST record coincided with these foraging bouts. While the FST records for F189 are the best available for any dolphin in the Sarasota area, it should be noted that F189 is not a long-term resident Sarasota Bay dolphin. There are no sighting records for her in the Sarasota Dolphin Research Program (SDRP) database before or after the FST deployment.

Observations of bottlenose dolphins in Sarasota Bay demonstrated that they use a variety of feeding techniques, many of which are specific to the prey species, habitat, or individual dolphin, and some of which appear to require maternal training (11). Nowacek (19) identified sequences of behaviors preceding prey capture. Direct capture of a fish is often immediately preceded by acceleration and/or a “pinwheel,” when a dolphin suddenly rotates around the midpoint of its body when it is side-swimming, presumably to re-orient toward an escaping prey item. “Fish whacking” also may occur immediately prior to prey capture, as a dolphin strikes a fish with its flukes, often propelling fish into the air (34). Earlier in the foraging sequence, back-and-forth head movements described as “scanning” may be used to search for or assess potential prey. Along the edges of seagrass meadows, “kerplunking,” which involves forcefully driving the flukes through the water’s surface, creating a geyser and bubbles, may be used to flush prey. Sarasota Bay dolphins also make use of structures such as seawalls and bridge pilings to limit the movements of their prey. Most feeding is done by individuals, capturing a single fish at a time. In some cases, multiple dolphins seem to coordinate their activities to limit the movements of fish schools.

Two methods were used to assess the timing of Sarasota Bay dolphin sound production as an indication of activity relative to time of day. The first method involved the first-ever 24-h deployment of a DTAG in May 2011on FB90, a 41-year-old female. Preliminary analyses indicated that she was acoustically active around the clock, although fewer whistles and echolocation buzzes were produced during late afternoon and early evening than at other times. FB90’s average signature whistle rate was 0.20/min during the day and 0.11/min at night (P. Tyack, personal communication, 8 July 2013).

Echolocation buzzes by FB90 occurred throughout the 24-h period and were clustered into bouts; however, it is not possible to say for certain that all of the buzzes came from the tagged animal. Average buzz rates were 0.97/min during both day and night (P. Tyack, personal communication, 8 July 2013). Findings from previous studies of Sarasota Bay dolphins suggest that echolocation is used more frequently for feeding than in any other context (18–20, 35, 36). If the occurrence of echolocation buzzes in recordings is a reasonable indicator of feeding activity, then the data from FB90 provide additional support for dolphins feeding around the clock. The acoustic patterns exhibited by this single dolphin are intriguing, but more DTAG deployments will be necessary to fully define sound production relative to time of day for individual Sarasota Bay dolphins, and to understand the behavioral contexts under which sounds are produced.

**Figure 7:**
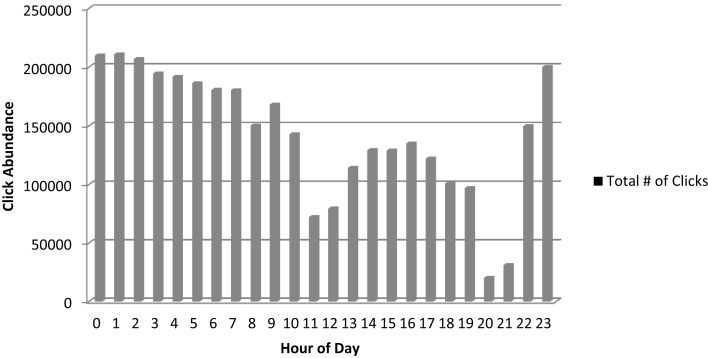
**Click occurrence at fixed hydrophone array during September 4–11, 2012 (0 h refers to the time period between 12 and 1 a.m.)**.

The second method involved deployment of a fixed hydrophone array, which collected recordings around the clock in an area of heavy Sarasota Bay resident dolphin use in Palma Sola Bay. Over the 1 week deployment period, 3,403,209 individual clicks were estimated to have occurred (Figure 7). Echolocation clicks were recorded throughout the day and night, but occurred most frequently at night to early morning, especially from about 23:00 to 03:00, with a secondary peak in abundance in late afternoon and early evening.

### Dolphin diet

Direct examination of stranded dolphin stomach contents, as well as DNA analyses of gastric samples and feces from live Sarasota Bay dolphins, indicate that these animals eat a variety of fish. These data also show the dolphins are selective about the prey they capture, and diets may change over time, probably in response to changes in prey availability (28–30, 37). During 1984–2006, stomach contents of 33 stranded Sarasota Bay dolphins were collected and 522 fish were examined. The eight most frequently occurring species or families of prey fish across the entire period, defined as appearing in at least five dolphin stomachs, and accounting for more than 84% of prey items, are listed in Table 2. The dominant prey fish were Sparids, especially pinfish (*Lagodon rhomboides*), and Gulf toadfish (*Opsanus beta*), accounting for a combined 72% of identified prey.

**Table 2:** **Summary of most frequently occurring fish in stomach contents of stranded Sarasota Bay bottlenose dolphins, 1984–2006**.

Prey fish	Are fish soniferous?	No. of stomachs with fish	No. of fish found	% Of total stomachs	% Of total fish
Sparids (including pinfish (*Lagodon rhomboides*), sheepshead (*Archosargus probatocephalus*), etc.)	No	21	199	63.6	38.1
Toadfish (*Opsanus beta*)	Yes	12	178	36.4	34.1
Pigfish (*Orthopristis chrysoptera*)	Yes	9	12	27.3	2.3
Spot (*Leiostomus xanthurus*)	Yes	7	16	21.2	3.1
Mullet (*Mugil cephalus*)	No	6	11	18.2	2.1
Clupeids (including menhaden [*Brevoortia* sp.), Atlantic thread herring (*Opisthonema oglinum*)]	No	6	11	18.2	2.1
Seatrout (*Cynoscion* spp.)	Yes	6	7	18.2	1.3
Ladyfish (*Elops saurus*)	No	5	7	15.2	1.3

List includes all fish found in at least 5 of 33 sampled dolphin stomachs, based on examination of 522 fish.

Barros and Wells (28) reported on the stomach contents of 16 Sarasota Bay dolphins stranded during 1984–1996, and identified a minimum of 15 prey fish species. Up to 54 individual prey fish were found in a single stomach, with common prey estimated to have been up to 28.5 cm long, weighing about 255 g. The most important species in terms of frequency of occurrence were pinfish, striped mullet (*Mugil cephalus*), pigfish (*Orthopristis chrysoptera*), and spot (*Leiostomus xanthurus*). The primary prey fish and observations of feeding both showed a strong association with seagrass habitat.

As a follow-up to Barros and Wells (28), analyses by Barros of stomach contents collected from 17 subsequent stranded Sarasota Bay dolphins during 1998–2006 were reported by Berens McCabe et al. (29). In total, 281 fish from 13 families and 22 species were identified. The four most abundant species were: Gulf toadfish, pinfish, ladyfish (*Elops saurus*), and spotted seatrout (*Cynoscion nebulosus*). Together, these four species accounted for 60% of the fish identified.

Barros et al. (37) suggested that the changes over time in the relative importance of different prey fish in the diets of the Sarasota Bay dolphins may have been related to changes in prey fish availability, as brought about by a state-wide commercial net fishing ban implemented in 1995. They compared the number of prey species found in dolphin stomachs before (1984–1995) and after (1998–2006) implementation of the net ban. The number of prey species found in stomachs increased from 14 fish species Pre-Net Ban to 33 species Post-Net Ban, and the prey diversity nearly doubled from an average of 2.4 prey taxa/stomach, Pre-Net Ban, to 4.3 prey taxa/stomach, Post-Net Ban. The change in diversity of diet coincided with a change in ^13^C and ^34^S isotopes in dolphin muscle, suggesting that the dolphins shifted from a primarily seagrass foraging habitat during the Pre-Net Ban period, to a broader foraging habitat, including open bays, during the Post-Net Ban period, as documented for some resident dolphins (11). It was suggested by Barros et al. (37) that changes may have been associated with a reduction in gillnet bycatch and recovery of at least some of the fish species incidentally impacted by the nets.

Sarasota Bay bottlenose dolphins select and consume some fish species disproportionately based on their availability within the dolphins’ range. In particular, sound-producing (i.e., soniferous) fish occurred more frequently in stomach contents from 1998 to 2006 than would have been expected given relative abundances in the study area (29). Of the 281 fish collected from dolphin stomachs during 1998–2006, 52% were soniferous species. Soniferous fish species account for half of the most commonly consumed prey listed in Table 2.

### Dolphin movements and dive patterns

The long-term resident bottlenose dolphins of Sarasota Bay concentrate their daily activities within a well-defined community home range (11). Some individuals, especially adult males, may move beyond the borders of the long-term community range for periods of time, and some individual core areas within the community range may shift over time, but the vast majority of resident dolphins demonstrate their fidelity to the community range throughout the year, for most of their lifetime (10–12, 34, 38).

Radio-tracking results from 1975 to 1976 indicated that bottlenose dolphins in Sarasota Bay are active both night and day, moving through a variety of habitats (10, 13). On occasion during some night radio-tracks, dolphins remained in one location for up to several hours, sometimes staying continuously at the surface (10). In general, there was no clear differentiation between habitats used during the day vs. at night.

**Figure 8:**
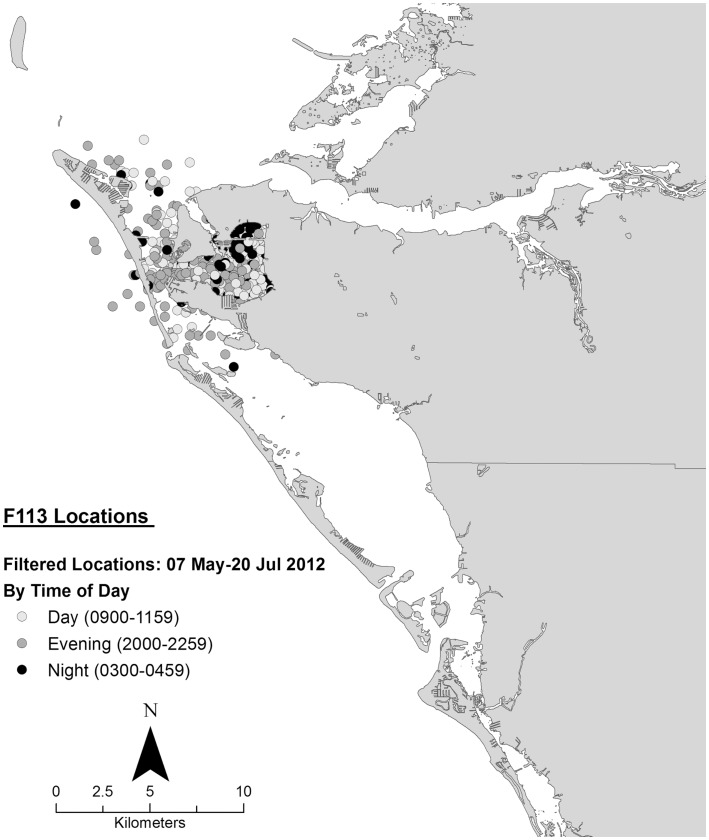
**Locations of bottlenose dolphin F113 relative to time of day during May–July 2012, from satellite-linked transmitter**.

**Figure 9:**
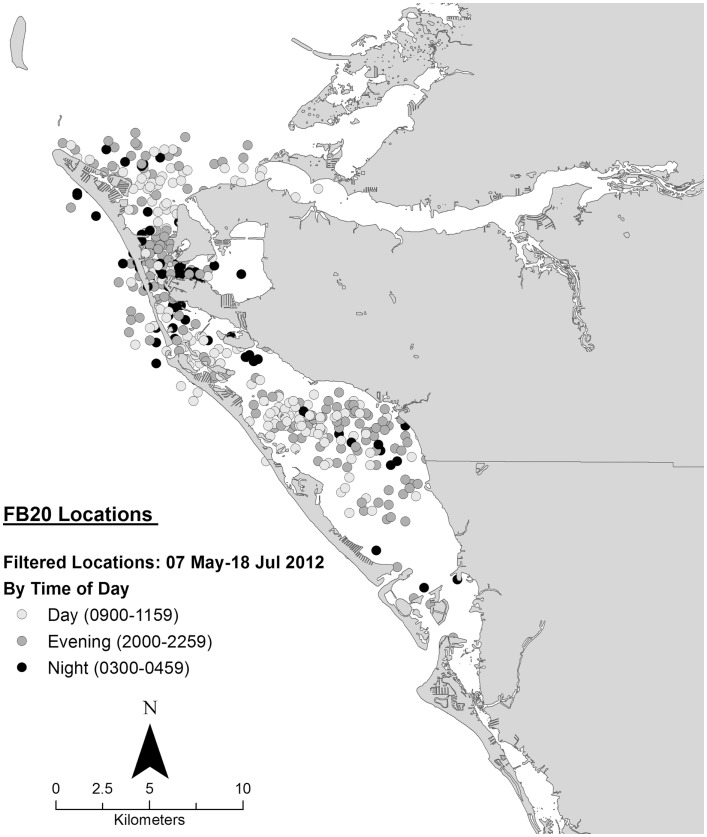
**Locations of bottlenose dolphin FB20 relative to time of day during May–July 2012, from satellite-linked transmitter**.

**Figure 10:**
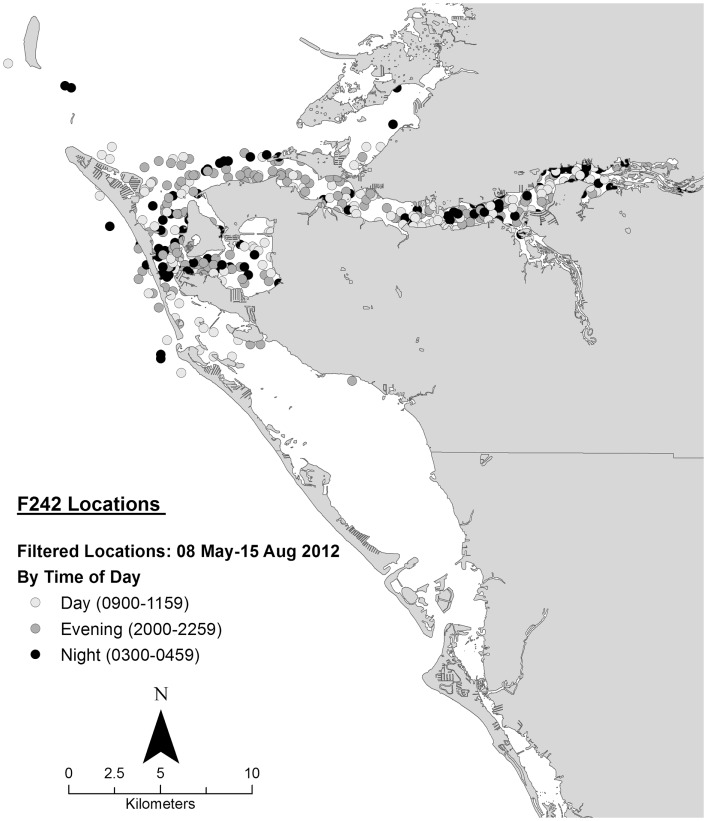
**Locations of bottlenose dolphin F242 relative to time of day during May–August 2012, from satellite-linked transmitter**.

Data collected on ranging patterns during 2012 support the findings from 1975 to 1976 of dolphins generally moving throughout the day and night within the home range of the resident community. Individual dolphins emphasized different portions, or core areas, of the community range (Figures 8–10). Adult female F113 remained primarily in Palma Sola Bay, Cortez, and Anna Maria Sound (Figure 8). Adult male FB20 ranged more widely, from the mouth of the Manatee River southward to southern Sarasota Bay (Figure 9). Adult male F242 emphasized the northern portion of the Sarasota community range, especially the waters of the Manatee River, Terra Ceia Bay, Anna Maria Sound, and Palma Sola Bay (Figure 10). All three overlapped in their extensive use of Anna Maria Sound. All three dolphins used all portions of their ranges during all times of day.

Observation of F189 in 2005 (21) showed her affinity to the Venice Inlet area and Little Sarasota Bay during a 2-day focal follow. During the first night, the dolphin spent several hours ranging back and forth within the inlet inside a 0.5 km^2^ area engaging in apparent foraging activity. Over the entire 41.2 h period, she swam a total distance of 136 km, yet ranged repeatedly over a linear stretch of only 17.5 km within the estuary.

Radio-tracking studies during 1975–1976, examining dive patterns for 10 tagged individuals, found hourly average dive durations of 30–40 s, with the longest recorded dive of 4 min 25 s (13). These early tracking studies did not find significant daily variation in diving patterns, perhaps because the shallow nature of the Sarasota Bay habitat (typically less than 4 m deep) precluded the need for deep or long dives (10, 13). Although insignificant with regards to their effect on average dive durations relative to time of day, on some occasions during night radio-tracks Sarasota Bay dolphins appeared to remain continuously at the surface (10).

In contrast, adult female F157, tagged with a satellite-linked TDR tag in 1990, exhibited significant dive duration differences among four 6-h periods of the day, along with variation in the mean percent of time spent submerged (32). During the “early morning” (02:11–08:10) F157 spent more time at the surface, averaged shorter dives, and was submerged less than during other times of day, suggesting the satellite-monitored dolphin may have been surface resting. F157 moved through deeper waters than Sarasota Bay residents, immediately north of the Sarasota Bay area.

**Figure 11:**
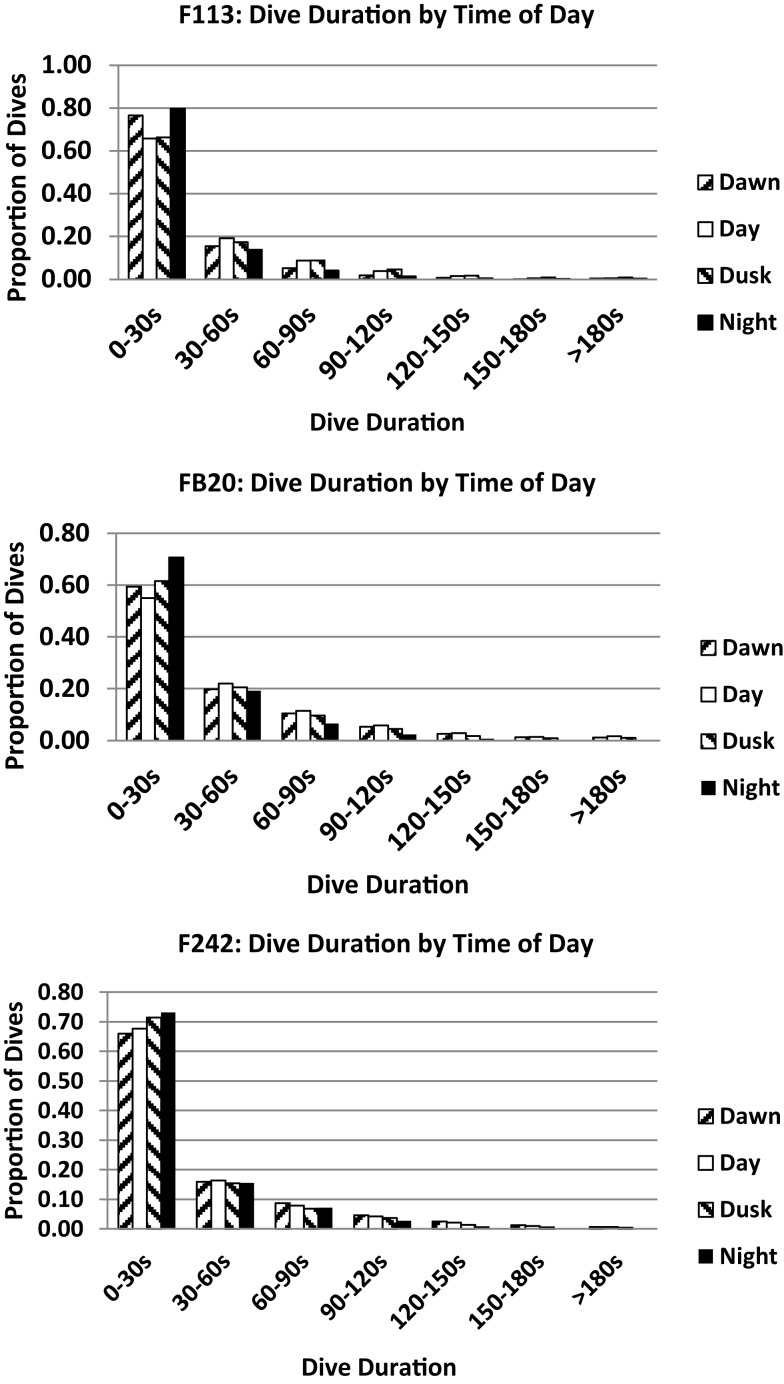
**Dive durations relative to time of day for bottlenose dolphin F113, FB20, and F242**.

Small but consistent variations in dive durations across the day were noted for the three dolphins tagged with satellite-linked transmitters in 2012 (Figure 11). Most dives were 0–30 s in duration, approximating the findings from 1975 to 1976. Few dives exceeded 150 s. The largest proportion of short dives (0–30 s) occurred during “night” (22:00–03:59) for all three individuals. This partially overlaps (02:11–03:59) the period of shorter dives by F157 (32), and the anecdotal night-time records of apparent surface resting during 1975–1976 tracking (10). The proportions of dives between 30 and 60 s were unchanged across the day for all three dolphins. The lowest proportions of dives >60 s occurred during “night.”

Irvine et al. (13) estimated travel speeds of about 2–5 km/h for bottlenose dolphins tracked during 1975–1976. Swimming speeds were measured during radio-tracking of Trac Pacs, by means of an onboard datalogger, or approximated from measurements of tracking vessel speed (21). Day-time swimming speeds averaged 5.15 km/h. During the night, swimming speeds averaged 5.26 km/h. The difference in swimming speeds between these time periods was not significant. Shippee et al. (21) identified intermittent, slow-speed, constant-direction swimming with rhythmic respiration rates and relaxed exhalations during both day and night for the two dolphins tracked during both of these periods. Shippee et al. (21) did not observe the deep, motionless sleep as noted for dolphins in some managed populations [e.g., (22)].

**Figure 12:**
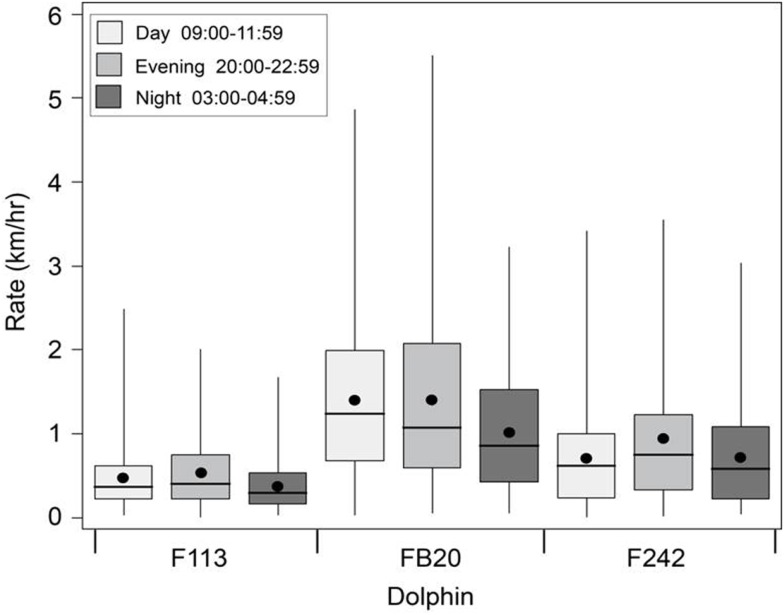
**Boxplots of travel rates for Sarasota Bay bottlenose dolphins tagged with satellite-linked transmitters during 2012**. Time-of-day categories (local) were defined by the tags’ pre-programed transmission windows (duty cycle). Boxes show interquartile ranges, dots, and lines respectively denote means and medians, and whiskers span minimum and maximum values.

Travel rates can also be measures of how much overall movement or displacement occurred during specific time periods, regardless of how fast the dolphin was swimming. We applied the continuous-time correlated random walk (CRAWL) approach (33) to the 2012 data from Sarasota Bay dolphins tagged with satellite-linked transmitters to regularize the tracking time interval and temper the effects of locational errors. On average, the dolphins traveled at a rate of about 0.9 km/h (Figure 12). Overall, FB20 had the highest travel rates, and F113 had the lowest. Travel rates for F113 could potentially have been affected by events occurring near the beginning of her tag deployment, including early pregnancy, entanglement of a fluke in fishing line, and association with a calf that was injured by an apparent boat collision; however, the impact of these factors on her movement patterns is unknown. In spite of apparent inter-individual differences in rates, all three dolphins exhibited consistent circadian patterns, with somewhat lower rates of movement during the night (03:00–04:59) transmission period. This night-time period of decreased travel rate at least partially overlaps the period of possible surface resting described by Mate et al. (32) and Scott et al. (10), providing further support for the general occurrence of a night-time rest period.

## Discussion

Based upon known risk factors for insulin resistance in humans, a variety of reasons have been proposed to explain why Sarasota Bay bottlenose dolphins may be less likely to have insulin resistance and metabolic syndrome compared to a managed group of dolphins (3). To further assess potential protective factors against metabolic syndrome in dolphins, age, meal size and frequency, diet, and work schedules and activity patterns were evaluated in the well-studied wild population of the long-term resident dolphin community in Sarasota Bay, Florida.

### Age

Older age is a risk factor for metabolic syndrome in humans, and as such, has been proposed as a similar risk factor in dolphins (2, 5). The managed population reported to have dolphins with metabolic syndrome has high annual survival rates, and an increasing number of dolphins living 40–50 years (39). Comparisons of age at death with Sarasota Bay dolphins suggest that the managed population studied by Venn-Watson et al. (3) is indeed older. For the managed population, age at death, as measured since 2003, is 32.6 years (40). In Sarasota Bay, roughly one third of dolphins that disappear are eventually recovered as carcasses. Considering the carcasses of residents recovered during 1993–2013, the average age at death was about 19.9 years. Further, similar to older humans, dolphins in the managed collection are more likely to have higher lipids and chronic inflammation on routine blood samples as they aged from 30 to 50 years old (41). Even when controlling for age, however, the managed dolphins still had higher insulin and lipids compared to Sarasota Bay dolphins, supporting that age is not the sole driver of insulin resistance and metabolic syndrome in dolphins. Further studies are needed to determine how and if old age influences dolphin metabolism.

### Meal size and feeding frequency

In humans, ingestion of smaller, more frequent meals (nibbling) can decrease postprandial insulin and glucose and improve glucose control (6). Similarly, feeding studies with dolphins have demonstrated increased insulin and glucose response correlated with increased amounts of fish eaten (42). Interestingly, this study reports that Sarasota Bay dolphins appear to nibble throughout the day and night; dolphins feed often, one to a few fish in a bout equivalent to a “meal,” spread over the entire day. Focal animal behavioral observations from boats and overhead video [e.g., (18–20, 23, 24)] show that Sarasota Bay dolphins feed at intervals during the day, presumably as need and opportunity present themselves. FST measurements indicated that dolphins in the Sarasota Bay area also feed at intervals at night (21). FST data from the Indian River Lagoon also indicated night-time feeding (21). Building on the assumption that echolocation is used more frequently for feeding than in any other context (18–20, 35, 36), further support for night-time feeding is drawn from acoustic recordings from DTAGs and a fixed hydrophone array, demonstrating the common use of echolocation at night.

In contrast, dolphins from the managed collection with reported cases of insulin resistance and metabolic syndrome typically consume fewer, larger meals fed over an 8-h period during daylight hours (4). If total daily food consumption is the same across both groups, it stands to reason that more meals through the day will mean smaller individual meals for the Sarasota Bay dolphins. Barros and Odell (43) found that stomach contents of stranded bottlenose dolphins weighed 0.683 kg, on average, but most contents weighed <0.500 kg. In contrast, managed dolphins fed 8–15 kg of fish each day in 3–5 feeding sessions might be expected to have 1.6–5.0 kg of food in their stomachs from one meal, about 3–10 times the amount for a wild dolphin. Thus, the Sarasota Bay dolphin feeding style alone may decrease their risk of insulin resistance and metabolic syndrome. The fact that stranded dolphins were in some cases ill and probably not eating normally needs to be considered for proper interpretation of this comparison.

### Fish-based nutrients

Nutrients found in fish, including n-3 fatty acids, can decrease the risk and help treat metabolic syndrome and insulin resistance in humans (9). Recommended diets from the American Heart Association and the Mediterranean Diet, both of which decrease the risks of metabolic syndrome, specifically include fish (44, 45). The possibility that there may be key nutritional differences between natural prey and commercially available food provided to the managed population was hypothesized to be a potential reason for the occurrence of metabolic disease in dolphins (3). Managed dolphins are fed a high-quality frozen – thawed fish diet including capelin, herring, mackerel, and squid (41). Free-ranging dolphins in Sarasota Bay and elsewhere are rarely observed eating dead fish, and then it is typically when they are provisioned by boaters (46, 47). Diets of wild bottlenose dolphins in Sarasota Bay and elsewhere typically do not include capelin or the species of herring fed to managed populations (28, 29, 43, 48). Instead, wild bottlenose dolphins consume a wide variety of live fish, and select some species disproportionately to their availability in the wild. In particular, unlike the food provided to managed dolphins, free-ranging bottlenose dolphins select soniferous fish (29, 43). The detection of soniferous prey by means of passive listening by dolphins has been demonstrated through field experiments in Sarasota Bay (36). Selective predation on soniferous fish reduces the need to use vision for foraging, opens up opportunities for dolphins to feed around the clock, and reduces the need to use energetically costly echolocation (36). Understanding key nutritional differences between fish eaten by Sarasota Bay and the managed dolphins may provide valuable insight into what and how fish protect against metabolic syndrome and insulin resistance in humans.

### Work schedules and activity patterns

Shift work can be either protective against, or a risk factor for, insulin resistance in humans, and circadian rhythm can cause day vs. night changes in insulin sensitivity (7, 8). Venn-Watson et al. (3) suggested that differences between the work schedules of the managed dolphins and their natural circadian rhythm may be among the reasons for the occurrence of metabolic disease. Sarasota Bay dolphins are active both during the day and at night, interspersing bouts of feeding, traveling, socializing, and idling or resting. This pattern of activity for bottlenose dolphins has been suggested by a number of authors [summarized by (49)], but until now a synthesis of data describing the activities of a full day in the life of members of a dolphin community has been lacking. The unique, long-term, natural laboratory situation in Sarasota Bay is providing an opportunity to compile information from a variety of sources to try to define a dolphin’s day and some of the factors, such as prey, that help to define it (12). Data from a variety of electronic tags, systematic behavioral observations, acoustic monitoring, and telemetry are providing new information on dolphin movements, dive patterns, quantification of activities and their cycles, and identification of feeding events relative to time of day.

The picture that emerges from consideration of the diverse data sets available from Sarasota Bay dolphins is one of animals that remain active throughout the day and night. Individuals vary in activity patterns, including timing of feeding peaks, foraging strategies used or prey items selected, and amount of overall time spent foraging. While their level of activity and movement may vary over the course of a day, the dolphins of Sarasota Bay are on the move throughout the 24-h period, alternating periods of activities such as traveling, foraging, socializing, and resting. Their movements do not place them at specific predictable locations at particular times of day, suggesting that other factors such as prey availability or social factors may play a larger role than strictly geography in guiding their ranging patterns during the day. In contrast, managed dolphins are likely most active during the normal work day when they take part in training, research, and husbandry sessions. Further studies are needed to truly measure differences in activity levels between dolphin populations to assess the role of the duration and types of activities on dolphin metabolism.

Several lines of evidence, including direct tracking and observations as well as remote tracking, suggest that dolphins may enter into rest periods at night and during the day. Unlike some dolphins in managed populations, the Sarasota Bay animals continue to move while resting, and do not enter into a motionless deep sleep at the surface during the night (21). These observations are consistent with the dolphin’s hemispheric sleep, which allows them to rest one side of the brain at a time and remain vigilant (50). During night-time rest periods Sarasota dolphins move more slowly and quiescently, and spend more time at or near the surface, engaging in briefer dives than at other times of the day. The deep, motionless surface rest of dolphins in managed populations may be an artifact of the captive situation, including the absence of predators. In one behavioral study of two bottlenose dolphins returned to Tampa Bay after 2 years in a research facility, it was demonstrated that dolphins engaged in traveling significantly more when in the wild than in the pool (51). Day-time rest periods for Sarasota dolphins do not appear to involve as much time at the surface, instead involving slow movements and absence of other activities. Also, the Sarasota Bay bottlenose dolphins continue to produce whistles and echolocation throughout the day and night. As shown from the DTAG and the fixed acoustic recorder, they do not reduce their sound production in a significant and predictable manner during well-defined, multi-hour daily resting periods. In contrast, some dolphin species, such as Hawaiian spinner dolphins (*Stenella longirostris*), clearly engage in extended resting periods. Spinner dolphins rest quietly in shallow bays for hours during the daytime, and become much more acoustically active as they move offshore to feed overnight (52).

The activity patterns of the Sarasota Bay dolphins can be considered representative of bottlenose dolphins at least along the west coast of Florida, and probably more broadly. The activity budgets of the Sarasota Bay dolphins, while varying by individual, overall were roughly consistent with those of dolphins studied in adjacent populations to the north, in Tampa Bay (53), and to the south, in Pine Island Sound (54). Waples (23) noted that in spite of some differences among the three study sites in proportions of time spent, the overall order of importance was generally consistent across all three sites: traveling > milling > feeding > socializing > resting.

Findings from field research on the biology, behavior, and ecology of Sarasota Bay dolphins demonstrated an empirical basis for several of the hypotheses proposed by Venn-Watson et al. (3) as potential reasons for the occurrence of metabolic disease in the well-studied managed dolphin population with insulin resistance and metabolic syndrome. Potential protective factors against metabolic syndrome and insulin resistance in Sarasota Bay dolphins may include younger age, specific fish nutrients in their diverse diet, eating small, frequent meals, and remaining active throughout the day and night as part of their natural circadian rhythm. Small, high-protein fish-based meals spread over 24 h and interspersed with periods of exercise may contribute to the absence of metabolic syndrome and insulin resistance among Sarasota Bay dolphins. Taken together or separately, these differences between managed and free-ranging dolphins provide the basis for better understanding insulin resistance, metabolic syndrome, and fatty liver disease in dolphins, as well as possibly helping to prevent or treat these conditions in humans.

## Author Contributions

Randall S. Wells provided data from the early years of the SDRP activities, led the more recent tagging and tracking efforts, synthesized the data from all sources, and drafted most of the manuscript. Katherine A. McHugh is responsible for current SDRP behavioral research and contributed data from recent behavioral studies. David C. Douglas performed travel rate analyses on recent data from dolphins tagged with satellite-linked transmitters. Steve Shippee provided data from FST research, tracking, and behavioral studies. Elizabeth Berens McCabe provided data to place dolphin prey selection into perspective. Nélio B. Barros (deceased) performed all of the stomach content analyses reported here. Goldie T. Phillips provided findings from a fixed hydrophone array.

## Conflict of Interest Statement

The authors declare that the research was conducted in the absence of any commercial or financial relationships that could be construed as a potential conflict of interest. The Guest Associate Editor, Stephanie Venn-Watson declares that, despite having collaborated with the author Randall Wells, the review process was handled objectively and no conflict of interest exists.
